# Randomised controlled trial of a psychiatric consultation model for treatment of common mental disorder in the occupational health setting

**DOI:** 10.1186/1472-6963-7-29

**Published:** 2007-02-27

**Authors:** Christina M van der Feltz-Cornelis, Jolanda AC Meeuwissen, Fransina J de Jong, Rob Hoedeman, Iman Elfeddali

**Affiliations:** 1 Program Diagnosis and Treatment, Trimbos Instituut, Netherlands Institute of Mental Health and Addiction, PO Box 725, 3500 AS Utrecht, The Netherlands; 2 Institute for Research in Extramural Medicine, VU Medical Centre, Amsterdam, The Netherlands; 3 Department of Psychiatry, VU Medical Centre, Amsterdam, The Netherlands; 4 University Medical Centre Groningen, University of Groningen, The Netherlands; 5 ArboNed Utrecht, The Netherlands; 6 University of Maastricht, Maastricht, The Netherlands

## Abstract

**Background:**

Common mental disorders are the most prevalent of all mental disorders, with the highest burden in terms of work absenteeism and utilization of health care services. Evidence-based treatments are available, but recognition and treatment could be improved, especially in the occupational health setting. The situation in this setting has recently changed in the Netherlands because of new legislation, which has resulted in reduced sickness absence. Severe mental disorder has now become one of the main causes of work absenteeism. Occupational physicians (OPs) are expected to take an active role in diagnosis and treatment, and seem to be in need of support for a new approach to handle cases of more complex mental disorders. Psychiatric consultation can be a collaborative care model to achieve this.

**Methods/design:**

This is a two-armed cluster-randomized clinical trial, with randomization among OPs. Forty OPs in two big companies providing medical care for multiple companies will be randomized to either the intervention group, i.e. psychiatric consultation embedded in a training programme, or the control group, i.e. only training aimed at recognition and providing Care As Usual. 60 patients will be included who have been absent from work for 6–52 weeks and who, after screening and a MINI interview, are diagnosed with depressive disorder, anxiety disorder or somatoform disorder based on DSM-IV criteria. Baseline measurements and follow up measurements (at 3 months and 6 months) will be assessed with questionnaires and an interview. The primary outcome measure is level of general functioning according to the SF-20. Secondary measures are severity of the mental disorder according to the PHQ and the SCL-90, quality of life (EQ-D5), measures of Return To Work and cost-effectiveness of the treatment assessed with the TiC-P. Process measures will be adherence to the treatment plan and assessment of the treatment provided by the Psychiatric Consultant (PC) in both groups.

**Discussion:**

In the current study, a psychiatric consultation model that has already proved to be effective in the primary care setting, and aimed to enhance evidence-based care for patients with work absenteeism and common mental disorder will be evaluated for its efficacy and cost-effectiveness in the occupational health setting.

## Background

Common mental disorders, i.e. depressive disorder, anxiety disorder and somatoform disorder, are the most prevalent of all mental disorders, with the highest burden in terms of work absenteeism and utilization of health care services [1-3]. In the case of depressive disorder, 80% of the costs are due to production loss [4]. People with major depressive disorder are absent from work 8 to 9 times as often as people without this disorder[5,6]. Mental problems account for 30% of disability leave, and in the majority of cases the employees have never been diagnosed or treated by a psychiatrist[7]. Complaints of the musculoskeletal system account for another 40%, and are considered to be possibly unrecognized somatoform, depressive and anxiety disorder[8]. Anxiety disorders such as generalized anxiety disorder and panic disorder, are also highly prevalent, and have a high societal impact[9]. Moreover, on average, absence from work due to mental disorders has a longer duration than absence caused by physical illness[10,11].

Guidelines for evidence-based treatments are available for depressive disorder, anxiety disorder and somatoform disorder[12-15,30]. However, for patients with these disorders in the primary care setting there is an inadequate offer of diagnostic and therapeutic help[16]. They often do not receive treatment, and present in the primary care setting with medically unexplained symptoms, thus burdening the General Practitioner (GP) and the health care setting in general with numerous visits and referrals to medical specialists[17-20]. The GP is misdirected by the combination of the patient's presentation with these symptoms and the fact that he has to meet competing demands in his practice[21]. If a mental disorder is recognized, the feasibility of these guidelines appears to be low, because many GPs feel incapable of delivering the cognitive behavioural interventions described in the guidelines, and also because of frequent care avoidance by the patients[22,23]. Therefore, care as usual (CAU) often implies fragmented patient-led care that is not cost-efficient, including referral to medical specialists to exclude somatic disease. This is a pity, because most of these patients can be treated effectively[24-27] in the primary care setting, if recognition and treatment is enhanced.

In the occupational health setting, that can be considered to have similar characteristics in terms of prevalence, diagnosis and treatment of mental disorders, as the primary care setting, a similar situation exists, but less attention has been paid to the diagnosis and treatment of common mental disorders. Guidelines for mental disorders in occupational health have been developed by the Dutch Association for Occupational Health (NVAB): guidelines for mental complaints[28], and, supplementary to the multidisciplinary guidelines for the diagnosis and treatment of depression[29], a module for depression and work. Guidelines have also been developed for the treatment of somatoform disorder in the occupational setting[30]. The NVAB guidelines for mental complaints[28] focus on stress and adjustment disorders in relation to the process of returning to work, functioning and the treatment of work-related problems. However, adequate recognition, treatment and criteria for the referral of patients for specialist treatment of mental disorders are not an important part of the guideline.

The implementation of the NVAB guidelines so far has led to a reduction in minor mental problems and in short-term work absenteeism. However, the amount of mental problems leading to disability leave remains high, indicating that occupational physicians (OPs) are still not accustomed to the diagnosis and treatment of mental disorders. In the Dutch legislation, responsibility for medical treatment is separated from responsibility for guidance in sickness absence and return to work (RTW). Only recently the OPs in the Netherlands have been enabled by law to refer employees to curative medical specialists if such treatment for a present mental disorder is needed for good functioning or RTW. Very little research has yet focussed on interventions for patients with common mental disorders, specifically aimed at RTW[3,31]. For many years it was assumed that recovery from symptoms would automatically lead to a recovery of functioning at work. However, it appears that focussing on functioning in the work setting contributes to a faster and a more lasting RTW in patients with mental disorders[32]. Thus, treatment of common mental disorder should not only focus on symptoms, but also on work-related functioning. This implies a broadening of the focus from the individual patient to the patient and the work context. Recovery should therefore also include the work setting: patients resume their work as soon as possible and are supported in this by their therapist [24]. For example, in a study carried out by Van der Klink et al. [25] among patients with adjustment disorders, in which the intervention was provided by OPs, the intervention had a shorter duration of sick leave than the control group. Therefore, in the present study we try to deal with sickness absence by embedding the treatment in the occupational setting and by including an intervention provided by the OP, after a consultation with a psychiatrist. Stimulating patients in RTW requires an active approach that encompasses specific interventions tailored to the needs created by the specific common mental disorder that the patient suffers from.

To summarize, in the present study design the following elements are central: training of OPs in the diagnosis and treatment of employees with depressive disorder, anxiety disorder or somatoform disorder; supportive psychiatric consultation aimed at the formulation of a diagnosis and a treatment plan, including suggestions for RTW adapted to the specific needs of the patients due to their specific disorder; and training of the consultant psychiatrists to provide not only a diagnosis and a treatment plan, but also to provide suggestions for successful strategies aimed at the improvement of work functioning.

## Methods/Design

### Objectives

The primary aim of the present study is to test the effectiveness of psychiatric consultation embedded in a training program for OPs, aimed at the diagnosis and treatment of common mental disorders in employees who are on sick leave, with a focus on work functioning. The secondary aim is to estimate the cost-effectiveness of the intervention.

### Hypothesis

In a randomized controlled study comparing psychiatric consultation with CAU provided by the OP, patients in the intervention group will improve in terms of general functioning, RTW, severity of the mental disorder and quality of life.

### Study design

The study is a two-armed cluster-randomized pragmatic clinical trial with randomization among the OPs. This intervention cannot be blinded, so the OPs might also use the techniques that they learned in the intervention to treat control patients. Randomization among OPs eliminates the danger of such contamination, and thus dilution of the effect. The outcome parameters will be measured by a blinded research assistant. This procedure has already been reported in detail elsewhere[33].

### Recruitment of OPs and consultant psychiatrists

The study has been designed and will be carried out in co-operation with ArboNed, and Arbounie, two companies providing company medical care. Together they cover almost half of the working population in the Netherlands. Within these two companies, 40 OPs from four offices in two regions will be recruited. Four consultant psychiatrists will also be recruited.

### Recruitment of patients

The aim is to include employees who have been absent from work for a minimum of 6 weeks and a maximum of 52 weeks, do not plan to resume work within the coming 6 weeks, and have been screened on either the Patient Health Questionnaire (PHQ) or the Whitely Index.

All patients who visited an OP within the past 6 months will be selected from the medical files, and will receive a letter describing the purpose of the study, together with an informed consent form for the screening procedure and the baseline questionnaires. Patients visiting an OP during the inclusion period will also be invited to participate in the study. They will also receive the informed consent form, together with the baseline questionnaires.

Patients will be included if they reach a cut off score of >15 on the PHQ9[34] or > 3.5 on the Whitely Index[35] and have given informed consent for participation in the study. The Mini-International Neuropsychiatric Interview (MINI.)[36,37] will be administered for the classification of symptoms according to DSM-IV[38]. In the intervention group, the patients selected in this way will have a consultation with a psychiatrist in addition to the treatment provided by the OP; in the control group the patients receive CAU provided by their OP.

### Exclusion criteria

**Figure 1:**
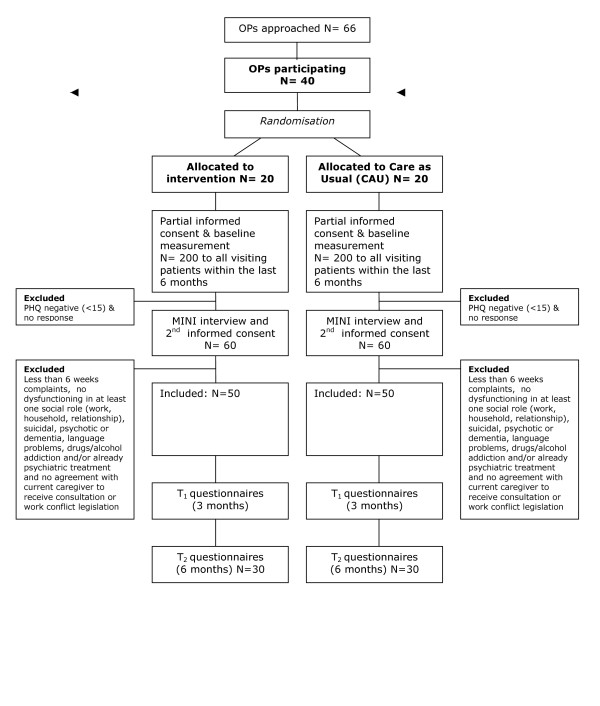
Flowchart of patients.

Patients will be excluded from the study if they are suicidal, psychotic or suffering from dementia, have insufficient knowledge of the Dutch language to fill in the questionnaires, or are addicted to drugs or alcohol. Patients who are already receiving psychiatric treatment can be included if mutual agreement is achieved with their current care-giver. Patients will also be excluded if they are involved in legislative procedures for unemployment compensation. (See Figure 1 for a flowchart of the participants.)

### Intervention

#### Training

All OPs will receive training in the diagnosis and treatment of common mental disorder in three sessions. They will be trained in the use of tools for screening and diagnostics, and cognitive-behavioural and reattribution techniques. The psychiatrists will be trained to make a diagnosis, and a treatment plan, and to formulate suggestions aimed at RTW specifically tailored to the patient's mental disorder. The psychiatrists will see the patient once and make a written report to the OP by consultation letter. A psychiatrist and an OP from the research group will provide the training.

#### Treatment in the intervention group

The care provided in the intervention group will follow a collaborative care approach: patient-tailored care executed within a team of the OP, the patient, the consulting psychiatrist, and in some cases the General Practitioner (GP). According to a previously developed method[20], a treatment plan will be formulated by the consultant psychiatrist, together with the patient. This will be specifically tailored to the diagnosed common mental disorder, with a focus on RTW. The OP will be informed about the treatment recommended by the consultant psychiatrist. Subsequently, the OP will co-ordinate the care and evaluate the treatment steps. During the consultation, the psychiatrist will make an inventory of treatment that has been offered to the patient before, and will decide, together with the patient, whether more intensive treatment is needed. This depends on the severity of symptoms, the perceived well-being of the patient, and on the level of general functioning according to the GAF score on Axis V of the DSM-IV[38] a method that has been described elsewhere[39].

#### Treatment in the control group

Half of the OPs will be randomized to the control group, and their patients will receive CAU. The actual content of the CAU (e.g. medication, number of contacts with OP, GPs and specialists) will be assessed according to the Scale for Medical Utilisation of Health Services[40].

### Outcome parameters

#### 1. Primary outcome measure

The primary outcome measure will be the level of functioning, as assessed with the SF-20[41-43].

#### 2. Secondary outcome measures

The severity of the symptoms will be measured with the PHQ[35] and the SCL-90[44]. Quality of life will be assessed with the EQ-5D[45,46] and the SF-20, both of which are validated tools for the assessment of general health-related quality of life.

In addition to the evaluation of improvement in general functioning, an attempt will also be made to assess the cost-utility of psychiatric consultation compared to CAU. Therefore, an estimate will be made of the direct medical costs and productivity costs. The necessary data collected by means of the Trimbos/*i*MTA questionnaire for Costs associated with Psychiatric Illness (TiC-P)[47,48].

Additional outcome measures will be the MVI-20, a questionnaire assessing fatigue[49] and the ALCOS-12 that assesses general competence, resilience and initiative in an employee[50].

Process measures will be satisfaction of the employee with the OP (VAS) and compliance with the advice given by the consultant psychiatrist.

Baseline measurements will take place before inclusion (T_0_), and follow up measurements will take place at 3 months with the TiC-P and EQ-5D (T_1_) and at 6 months (T_2_).

### Sample size

In the present study, there will be cluster randomization among the OPs, and Multi Level Analysis (MLA) will be performed with OPs at the first hierarchical level and patients at the second level. The SF-20 will be the primary outcome measure for the power calculation, which will be based on the design effect. This is the factor needed to enlarge the total sample size that would be reached the same standard error that would be reached using randomization between patients and a General Linear Model (GLM) analysis in the sample. The formula is[51]: **Design effect = 1+(n-1)ρ_1_**

n = the mean sample size at the second hierarchical level (patient level in this instance) and ρ_1 _= the intra-class correlation

If a GLM with repeated measures would be used in a study with randomization among patients, with a variance of 1.0, then a sample size of 2 × 10 would be needed for a power of 0.90. A variance of 1.0 as intraclass correlation would be acceptable as a presumption if the contrast between the two groups would be rather high, i.e. if the CAU that was provided was different to the consultation intervention provided in the different practices. We presume that this is the case, because psychiatric consultation in addition to OP care, as in the present study, is a new method for the Netherlands that differs substantially from normal standards of care [20]. Under these assumptions, in a MLA study such as this one, with an SD of 1.0, and a mean number of 3 patients per OP, this would result in a design effect of 3, implying that N should be multiplied by 3, compared to the number needed for a power of 0.90 in patient-randomised GLM analysis. If in such a study a standardized difference of 1.0 was detected, a sample size of 2 × 30 completers would be needed in order to detect a clinically relevant significant improvement of 25%, compared to the spontaneous recovery in the control group on the SF-20. This is the sample size that we aim to achieve in this study[52,53].

### Analysis

Intention-to-treat analysis will be performed by means of MLA, with OPs in the primary hierarchical level. Subsequently, at the secondary hierarchical level, patient characteristics influencing the outcome will be analysed. If differences in outcome are found in MLA, the effect size will be estimated by Chi-square analysis and described in Cohen's d[54]. To control for possible skew ness in the randomized groups, as far as the distribution of confounders is concerned, propensity scores will be calculated[55]. Possible confounders, such as age, gender, history of mental disorder, history of treatment, fatigue, resilience, and satisfaction and compliance of the employee, will be entered as variables in the analysis.

### Time-frame of the study

The preparatory period will be 6 months. Subsequent to the approval of the Medical Ethics Committee, the OPs will be recruited and the psychiatrists will be trained. The inclusion and intervention phase will take 12 months. The follow-up phase will be 12 months, and data-analyses will last for 6 months. The total duration of the study will be 3 years.

### Ethical principles

The study has been planned, and will be executed in accordance with the principles laid down in the Helsinki declaration (Edinburgh, Scotland amendment, October 2000). Participation in the study will be voluntary, and written informed consent will be obtained. Patients will be explicitly informed of the fact that they can withdraw their consent to participate at any time, without specification of reasons and with no negative consequences for their future medical treatment. Patients who wish to withdraw from the study will receive CAU. The study has been approved by the Medical Ethics Committee of the METiGG, Kamer Zuid protocol 5.127.

## Discussion

### Strengths and limitations

One limitation of the study might be that the organization of care in companies could be a hazard, because this sector is under pressure to work more and more efficiently. It might also be difficult to establish regular contact between OPs, consultant psychiatrists and GPs, because OPs generally work supraregionally. Specific attention will be paid to limitations in implementation due to regional differences.

A strength of the study might be that early consultation could be very helpful to enhance early RTW, and thus might also improve recovery from a mental disorder. By embedding the psychiatric consultation in the company care setting, timely access to specialist care will be improved and discontinuity of care will be avoided.

## Competing interests

The author(s) declare that they have no competing interests.

## Authors' contributions

CFC is principle investigator, daily supervisor, trainer, and psychiatric consultant. FJJ contributes as researcher and co-ordinator, and performs the MINI interviews. RH is OP co-ordinator, trainer and researcher. JM contributes as a researcher. IE performs the MINI interviews. All participants have contributed their expertise and have read and approved the final version of the article.

## Pre-publication history

The pre-publication history for this paper can be accessed here:



## References

[B1] Murray CJ, Lopez AD (1997). Alternative projections of mortality and disability by cause 1990–2020: Global Burden of Disease Study. Lancet.

[B2] Bijl RV, Ravelli A, Van Zessen G (1998). Prevalence of psychiatric disorder in the general population: results of The Netherlands Mental Health Survey and Incidence Study (NEMESIS). Soc Psychiatry Psychiatr Epidemiol.

[B3] Dutch Association for Occupational Health (2005). Module Depression and work. [In Dutch: Nederlandse Vereniging voor Arbeids- en Bedrijfsgeneeskunde (NVAB) Module Depressie en Arbeid].

[B4] Smit F, Willemse G, Koopmanschap M, Onrust S, Cuijpers P, Beekman A (2006). Cost-effectiveness of preventing depression in primary care patients: randomised trial. Br J Psychiatry.

[B5] Bijl RV, Ravelli A (2000). Psychiatric morbidity, service use, and need for care in the general population: results of The Netherlands Mental Health Survey and Incidence Study. Am J Public Health.

[B6] Kruijshaar ME, Hoeymans N, Bijl RV, Spijker J, Essink-Bot ML (2003). Levels of disability in major depression: findings from the Netherlands Mental Health Survey and Incidence Study (NEMESIS). J Affect Disord.

[B7] Prins R, Van der Burg C, Heijdel W (2005). Final Report SubCommittee for mental problems. [In Dutch: Eindrapport Subcommissie psychische klachten].

[B8] Mergl R, Seidscheck I, Allgaier AK, Moller HJ, Hegerl U, Henkel V (2006). Depressive, anxiety, and somatoform disorders in primary care: prevalence and recognition. Depress Anxiety.

[B9] Leon AC, Olfson M, Portera L (1997). Service utilization and expenditures for the treatment of panic disorder. Gen Hosp Psychiatry.

[B10] Brouwers EP, Tiemens BG, Terluin B, Verhaak PF (2006). Effectiveness of an intervention to reduce sickness absence in patients with emotional distress or minor mental disorders: a randomized controlled effectiveness trial. Gen Hosp Psychiatry.

[B11] Comes L, Van Eekeren A, De Leeuw F, Meekes M, Van de Plassche H, Van Santen A (2005). Return to Work Scenario for trainers [In Dutch: Terugkeer naar het werk Draaiboek voor trainers].

[B12] National Steering Group for Multidisciplinary Guidelines Development in Mental Health (2003). Multidisciplinary Guidelines anxiety disorderders. Guidelines for the diagnosis, treatment and monitoring of patients with an anxiety disorder [In Dutch: Landelijke Stuurgroep Multidisciplinaire Richtlijnontwikkeling in de GGZ Multidisciplinairerichtlijn angststoornissen 2003: richtlijn voor de diagnostiek, behandeling en begeleiding van volwassen clienten met een angststoornis.

[B13] Van Wamel A, Verburg H, Meeuwissen J, Voordouw I, Van de Velde V (2005). Landelijk basisprogramma angststoornissen [Dutch].

[B14] Terluin B, Van Heest FB, Van der Meer K, Neomagus GH, Hekman J, Aulbers LPJ, Starreveld J, Grol M (2004). Dutch College of General Practitioners Guideline for Anxiety Disorder. [In Dutch: NHG-Standaard Angststoornissen]. HA & W.

[B15] Van Marwijk HWJ, Bijl D, Van Gelderen M, De Haan M (2003). Dutch College of General Practitioners Guideline for Depression, first revision [NHG-Standaard Depressieve stoornis (depressie). Eerste herziening. In Dutch]. HA & W.

[B16] Ormel J, Bartel M, Nolen WA (2003). [Undertreatment of depression; causes and recommendations]. [Dutch]. Ned Tijdschr Geneeskd.

[B17] Bodenheimer T (2005). Helping patients improve their health-related behaviors: what system changes do we need?. Dis Manag.

[B18] Simon GE, Von Korff M, Barlow W (1995). Health care costs of primary care patients with recognized depression. Arch Gen Psychiatry.

[B19] Van der Feltz-Cornelis CM, van Oppen P, Adèr H, van Dyck R (2006). Randomised Controlled Trial of a Collaborative Care Model with Psychiatric Consultation for Persistent Medically Unexplained Symptoms in General Practice. Psychother Psychosom.

[B20] Mayou RA, Bass CM, Bryant BM (1999). Management of non-cardiac chest pain: from research to clinical practice. Heart.

[B21] Nutting PA, Rost K, Smith J, Werner JJ, Elliot C (2000). Competing demands from physical problems: effect on initiating and completing depression care over 6 months. Arch Fam Med.

[B22] Van Boeijen CA, Van Oppen P, Van Balkom AJLM, Visser S, Kempe PT, Blankenstein N, Van Dyck R (2005). Treatment of anxiety disorders in primary care practice: a randomised controlled trial. Br J Gen Pract.

[B23] Katon W, Von Korff M, Lin E, Lipscomb P, Russo J, Wagner E, Polk E (1990). Distressed high utilizers of medical care. DSM-III-R diagnoses and treatment needs. Gen Hosp Psychiatry.

[B24] Roy-Byrne PP, Craske MG, Stein MB, Sullivan G, Bystritsky A, Katon W, Golinelli D, Sherbourne CD (2005). A randomized effectiveness trial of cognitive-behavioral therapy and medication for primary care panic disorder. Arch Gen Psychiatry.

[B25] Roy-Byrne P, Stein MB, Russo J, Craske MG, Katon W, Sullivan G, Sherbourne C (2005). Medical illness and response to treatment in primary care panic disorder. Gen Hosp Psychiatry.

[B26] Rollman BL, Belnap BH, Mazumdar S, Houck PR, Zhu F, Gardner W, Reynolds CF, Schulberg HC, Shear MK (2005). A randomized trial to improve the quality of treatment for generalized anxiety disorder and panic disorders in primary care. Arch Gen Psychiatry.

[B27] Katon WJ, Roy-Byrne P, Russo J, Cowley D (2002). Cost-effectiveness and cost offset of a collaborative care intervention for primary care patients with panic disorder. Arch Gen Psychiatry.

[B28] Dutch Association for Occupational Health (2000). Monitoring of employees with mental problems by the Occupational Physician [In Dutch: Nederlandse Vereniging voor Arbeids- en Bedrijfsgeneeskunde (NVAB) Handelen van de bedrijfsarts bij werknemers met psychische klachten Richtlijn voor bedrijfsartsen].

[B29] CBO, Trimbos-Instituut (2005). Multidisciplinary Guideline Depressive Disorder [In Dutch: Multidisciplinaire Richtlijn Depressie].

[B30] STECR (2006). Work map approach medically unexplained symptoms and somatisation [In Dutch: Werkwijzer aanpak lichamelijk onverklaarde klachten en somatisatie].

[B31] Nieuwenhuijsen K, Verhoeven AC, Bültmann U, Neumeyer-Gromen A, Van der Feltz-Cornelis CM (2006). Interventions to improve occupational health in depressed people. (Protocol). Cochrane Database of Systematic Reviews.

[B32] Schene AH, Van Weeghel J, Van der Klink J, Van Dijk FJH (2005). Mental disorders and work: treatment. [In Dutch: Psychische aandoeningen en arbeid: de interventies]. Psychische aandoeningen en arbeid.

[B33] Van der Feltz-Cornelis CM, Ader HJ (2000). Randomization in psychiatric intervention research in the general practice setting. Int J Methods Psychiatr Res.

[B34] Kroenke K, Spitzer RL, Williams JB (2001). The PHQ-9: validity of a brief depression severity measure. J Gen Intern Med.

[B35] Speckens AE, Spinhoven P, Sloekers PP, Bolk JH, Van Hemert AM (1996). A validation study of the Whitely Index, the Illness Attitude Scales, and the Somatosensory Amplification Scale in general medical and general practice patients. J Psychosom Res.

[B36] Van Vliet IM, Leroy H, Van Megen HJM (2000). De MINI-Internationaal neuropsychiatrisch interview: een kort gestructureerd diagnostisch interview voor DSM-IV en ICD-10 psychiatrische stoornissen [Dutch].

[B37] Sheehan DV, Lecrubier Y, Sheehan KH, Amorim P, Janavs J, Weiller E, Hergueta T, Baker R, Dunbar GC (1998). The Mini-International Neuropsychiatric Interview (M.I.N.I.): the development and validation of a structured diagnostic psychiatric interview for DSM-IV and ICD-10. J Clin Psychiatry.

[B38] American Psychiatric Association (1994). Diagnostic and statistical manual of mental disorders: DSM-IV.

[B39] Van der Feltz-Cornelis, Knispel A, Effedali I (2006). Demarcation primary and secundary health care for mental disorder [In Dutch: Afbakening eerste en tweede lijnszorg voor psychische stoornissen].

[B40] Van der Feltz-Cornelis CM (2002). Psychiatric consultation for patients with somatoform disorder in general practice. PhD Thesis.

[B41] Kempen GI, Brilma E, Heimerink JW, Drul J (1995). Measurement of general health with the MOS short Form for General Health Survey (SF-20) [In Dutch: Het meten van de algemene gezondheidstoestand met de MOS Short Form for General Health Survey (SF-20)].

[B42] Ware John EJ SF-36 Health update. The SF community. http://www.sf-36.org/tools/sf36.shtml.

[B43] Hoeymans N, Piacinet HS, Tijhuis MAR Health related quality of life. Aim and definition. What is quality of life and how is it measured? [In Dutch: Gezondheidsgerelateerde kwaliteit van leven. Doel en definitie. Wat is kwaliteit van leven en hoe wordt het gemeten? Nationaal Kompas Volksgezondheid. [Dutch]. http://www.nationaalkompas.nl.

[B44] Arrindell WA, Ettema JHM (1992). SCL-90 Guide for a multidimentional psychopathology indicator [In Dutch: Handleiding bij een multidimensionele psychopathologie indicator].

[B45] Euroqol group (1995). EQ-5D user guide.

[B46] Dolan P (1997). Modelling valuations for EuroQol health states. Med Care.

[B47] Hakkaart-Van Roijen L (2002). Manual Trimbos/iMTA questionnaire for costs associated with psychiatric illness (in Dutch).

[B48] Hakkaart-Van Roijen LH, Van Straten A, al M, Rutten F, Donker M (2006). Cost-utility of brief psychological treatment for depression and anxiety. Br J Psychiatry.

[B49] Smets EMA, Garssen B, Bonke B (1995). Measurement of fatigue with the multidimensional Fatigue-Index: a guideline [In Dutch: Het meten van vermoeidheid met de multidimensionele vermoeidheids-index (MVI-20): multidimensional fatique inventory: een handleiding (MVI-20)].

[B50] Bosscher RJ, Smit JH, Kempen GIJM (1997). General Competence Expectations in the Elderly: research into the psychometric characteristics of the General Competence Scale (ALCOS). [In Dutch: Algemene competentieverwachtingen bij ouderen: een onderzoek naar de psychometrische kenmerken van de Algemene Competentieschaal (ALCOS)]. Nederlands Tijdschrift voor de Psychologie.

[B51] Snijders T, Bosker R (1999). Multilevel Analysis An introduction to basic and advanced multilevel modelling.

[B52] Cochran WG (1977). Sampling techniques.

[B53] Keny SN, Bland JM (1988). Analysis of a trial randomised in clusters. BMJ.

[B54] Cohen J (1988). Statistical power analysis for the behavioral science.

[B55] Van der Feltz-Cornelis CM, van Oppen P, Adèr H, van Dyck R (2006). Collaborative care for Medically Unexplained Symptoms in General Practice. Outcome and Benefits for the General Practitioner. [Collaborative care voor lichamelijk onverklaarde klachten in de huisartsenpraktijk. Uitkomst en opbrengst voor de huisarts]. HA & W.

